# The Co-occurrence of Cleft and Clubfoot: A Case Report on a Rare Medical Condition

**DOI:** 10.7759/cureus.62196

**Published:** 2024-06-11

**Authors:** Jigna Kachaliya, Pallavi Daigavane, Khushi S Zanwar, Mrudula Shinde, Nishu Agarwal, Bhagyashri Chimote

**Affiliations:** 1 Dentistry, Sharad Pawar Dental College and Hospital, Datta Meghe Institute of Higher Education and Research, Wardha, IND; 2 Orthodontics and Dentofacial Orthopedics, Sharad Pawar Dental College and Hospital, Datta Meghe Institute of Higher Education and Research, Wardha, IND; 3 Prosthodontics, V.Y.W.S. Dental College and Hospital, Amravati, IND

**Keywords:** cleft lip and palate, deformities, syndromic association, congenital abnormalities, clubfoot

## Abstract

Cleft lip and palate (CLP) are complex deformities in craniofacial development that can range from isolated to syndromic presentations. This case presentation emphasizes the identification and early management of syndromic associations in patients with CLP. The report presents a unique case of a one-month-old female patient with complete unilateral CLP and clubfoot. The patient was comprehensively assessed, and a treatment plan was formulated. Presurgical nasoalveolar molding was done for the initial alignment of cartilages and alveolar bone. The treatment modalities for clubfoot are presented in the discussion. The following presentation emphasizes the characteristics of syndromic CLP and the importance of multidisciplinary therapy toward optimum patient care. This report underlines the role of coordinated efforts in managing the multifaceted needs of patients with complex congenital conditions to improve their overall well-being and quality of life.

## Introduction

The development of the craniofacial region is one of the most complex phases of embryonic development. Jaw and face deformities may result from any intervention with the fetus during this developmental stage. One of the most common malformations is cleft lip and palate (CLP), which stands out as one of the most common congenital anomalies [1]. Cleft palates are expected to occur in 1.42 live births out of every 1,000, while isolated cleft palates occur in one live infant out of every 2,000 [2]. The complex etiology of CLP has been thought to be the result of an interplay between hereditary and environmental factors. Genetic factors include syndromic and nonsyndromic clefts, while nongenetic factors include alcohol intake, smoking, maternal conditions, and stresses during pregnancy [3]. CLP can be classified into syndromic or nonsyndromic, depending on its presentation as an isolated anomaly or part of a particular pattern of malformation. Strong hereditary influences CLP in both kinds. Monogenic illnesses and chromosomal abnormalities are often the cause of syndromic variations. For example, Van der Woude syndrome, which causes approximately 2% of instances of syndromic CLP, is linked to abnormalities in the regulatory transcription factor 6 gene. That being said, nonsyndromic CLP is a complicated condition resulting from a combination of genetic and environmental predispositions [4].

CLP is one of the characteristics of over 300 disorders. Van der Woude syndrome, Pierre Robin syndrome, Treacher Collins syndrome, Van der Woude syndrome, and Cleidocranial dysostosis are common syndromes associated with CLP. Rarely occurring syndromes include orofacial digital syndrome, Apert syndrome, Sticklers syndrome, Mohr syndrome, Robert syndrome, etc.

According to research conducted by Rittler et al. [5], congenital talipes equinovarus (CTEV), also known as clubfoot, is a rare condition that manifests in approximately 13% of cases with syndromic cleft lip with or without palate, 20% of syndromic cases of cleft palate, and 13% of non-orofacial birth defects. For newborns with clubfoot, early intervention is crucial to preventing consequences like arthritis, calluses, foot infections, and decreased mobility. Not addressing clubfoot can lead to challenging gait patterns; therefore, taking care of your feet as soon as possible is critical to preserving ideal health. Clubfoot can be treated surgically or nonsurgically (using the Ponseti procedure).

The report's main goal is to get people with syndromes diagnosed and treated immediately. This can entail giving medical practitioners comprehensive instructions or suggestions regarding the significance of early diagnosis and treatment.

## Case presentation

**Figure 1:**
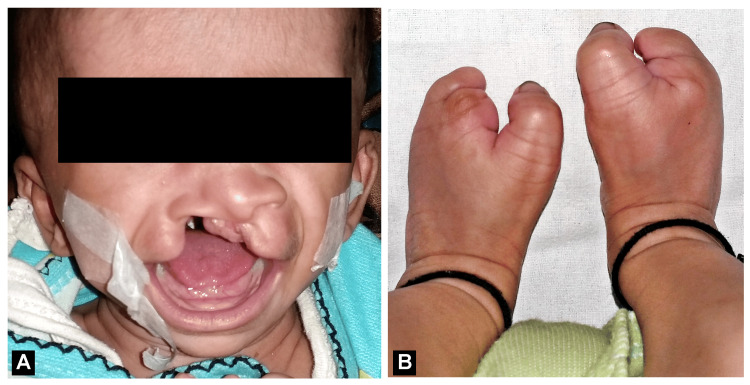
(A) Complete unilateral cleft lip and palate. (B) Clubfoot.

A one-month-old female infant with complete unilateral CLP was reported to the Department of Orthodontics and Dentofacial Orthopedics, Datta Meghe Institute of Higher Education and Research, Wardha, Maharashtra, India (Figure 1A). One defining feature of the infant (Figure 1B) was clubfoot, a disorder associated with one out of every 1,000 newborns with CLP.

A comprehensive intraoral, extraoral, and overall body examination was recommended for heart anomaly, presence of a Simonart's band, etc. A full body examination for other external parts of the body was done in detail, and no other anomaly except clubfoot was found. An echo was advised, and it was normal. The patient was then referred to the Smile Train unit within the Department for Presurgical Infant Orthopedics. The diagnosis of syndromic CLP with many related abnormalities was validated in light of the anomalies associated with the condition.

**Figure 2:**
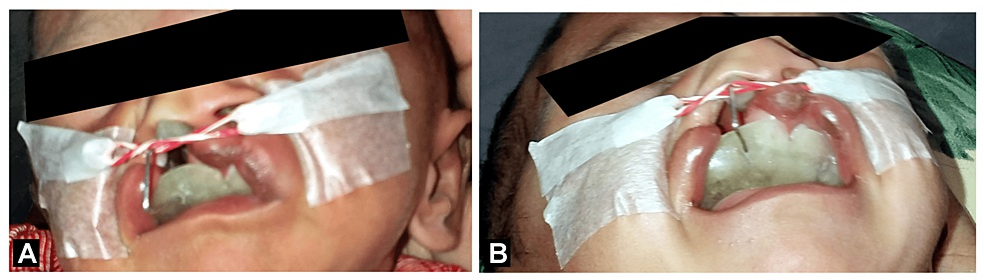
(A,B) Cleft lip and palate repair using PNAM PNAM: presurgical nasoalveolar molding

After the complete investigations and assessments, such as blood investigations and echo, a treatment protocol was decided for the patient. As the patient was one month old, this was the time for alveolar molding utilizing cartilage molding. Thereby, "presurgical nasoalveolar molding" (PNAM) was planned until lip repair. Grayson’s modified technique was used to mold the alveolar and nasal cartilage (Figures 2A, 2B).

The patient was recalled every week for nasal molding. The further protocol included lip repair at six to seven months of age and palatal repair at 14-18 months, followed by treatment for clubfoot when advised.

## Discussion

A cleft is an abnormal gap or opening in the upper lip, palate, or alveolus that results from an inborn abnormality. Many times, monogenic illnesses or chromosomal abnormalities cause syndromic manifestations in patients with CLP, known as syndromic clefts. Delayed tooth maturation, ectopic eruption, microdontia, taurodontism, natal and neonatal teeth, and enamel hypoplasia are dental issues linked to CLP [6]. Numerous difficulties may arise from the disorder, such as social difficulties, speech impediments, hearing loss, malocclusion, substantial facial abnormalities, serious psychiatric concerns, and decreased nursing, leading to failure to thrive [6]. Treatment for syndromic and nonsyndromic clefts differs; the former can be addressed with ordinary appliance therapy, early intervention with PNAM followed by surgical repair, expansion in mixed dentition, and fixed mechanotherapy in permanent dentition while the latter requires medical evaluation before treatment begins. Orofacial clefts are the most prevalent craniofacial birth anomalies and rank second to clubfoot in common birth defects [7]. Managing these conditions entails a comprehensive, multidisciplinary approach involving collaboration across various fields and specialties. The orthodontist should ensure that the patient is satisfied with the functional and aesthetic concerns and that the patient's aesthetics have not been compromised [8].

Clubfoot (CTEV) is a disorder marked by an anomaly in three dimensions that affects the leg, ankle, and feet, causing the foot and ankle joint to be positioned abnormally. As estimated in a 2018 study, its frequency is supposed to be 1 in 1,000 live births, according to Pavone et al. [9]. According to O'Shea and Sabatini, approximately 80% of CTEV cases are considered nonsyndromic, and their occurrence is complex, with many factors interplaying [10]. However, 20% of instances are syndromic.

Researchers have proposed several theories to explain the pathophysiology of congenital clubfoot, even though the condition's specific origin is unknown. The intrauterine position of the fetus, mechanical pressures inside the womb, disruptions in fetal development, viral infections, vascular and muscular problems, neurological abnormalities, irregularities in the development of bone structure, and genetic factors are just a few of the extrinsic and intrinsic factors examined by these theories [11].

The patient presented with a full unilateral CLP and clubfoot. The treatment approach for this patient would prioritize addressing the CLP initially, followed by treating the clubfoot. Correcting malformations of the CLP is one of the trickiest surgical problems.

The patient was treated by PNAM, followed by surgical intervention. With the PNAM method pioneered by Grayson and Shetye, precise alignment of the alveolus, lip, and nose was achieved. Its objective is to lengthen the columella and lessen the severity of the nasal deformity without requiring surgery, which was targeted with the appliance [12]. The PNAM appliance lessened the severity of clefts. This method shapes nasal cartilage using a specialized appliance for infants as young as six weeks old until cleft lip and nose surgery around five months. Its early use enhanced the appearance of cleft babies and positively impacted parental psychology, showing promising results in the cases [13]. This, in turn, would help surgeons achieve a more favorable and reliably stable outcome after the end of the procedure.

Previously, it was commonly believed that nonsurgical methods could not effectively correct idiopathic clubfoot and provide lasting results, leading most children to undergo extensive posteromedial and lateral release surgery. However, some children experienced lingering deformities, stiffness, and pain after surgery. This has raised interest in the favorable long-term outcomes of Ponseti and French nonsurgical treatments. While the Ponseti method entails manipulating and casting idiopathic clubfeet, the French method uses continuous passive motion, taping, and physiotherapy [14].

Before the widespread adoption of the Ponseti technique, medical practitioners experimented with various methods to address clubfoot, which often led to recurring problems such as stiffness and pain [15]. These methods, including Hippocrates' ancient suggestion of manipulation and bandaging, as well as more modern approaches involving casting and corrective tools, ultimately proved inadequate in providing lasting relief [16]. Surgery is usually indicated when adequate nonsurgical treatment fails to correct the deformities. McKay [17] emphasized the importance of abnormal horizontal calcaneal rotation in his presentation on "new concepts of and approach to clubfoot treatment." Simons [18] presented a complete subtalar release in clubfoot in 1985. His techniques and ideas were fundamentally similar to those of McKay. The complete subtalar release procedure of McKay and Simons would appear to represent the most complete single-stage release currently available for clubfoot.

## Conclusions

CLP is multifactorial and presents numerous extraoral and intraoral features that affect hard tissue, soft tissue, and functions. In any patient with CLP, the results are best achieved through a multidisciplinary approach. The report's primary objective is to emphasize the importance of presenting rare cases and prompt identification and care for CLP patients with other related malformations, clubfoot being one of them.

The case report highlights the complexity of congenital defects, including clubfoot and CLP. A thorough evaluation of the one-month-old female patient with both clubfoot and complete unilateral CLP was done, and the best fit treatment strategy was formulated. The treatment plan for the patient included PNAM for the initial alignment of cartilage and alveolar bone. In addition, some clubfoot treatment approaches have been discussed.
